# He-ion Irradiation Effects on the Microstructures and Mechanical Properties of the Ti-Zr-Hf-V-Ta Low-Activation High-Entropy Alloys

**DOI:** 10.3390/ma16165530

**Published:** 2023-08-09

**Authors:** Huanzhi Zhang, Qianqian Wang, Chunhui Li, Zhenbo Zhu, Hefei Huang, Yiping Lu

**Affiliations:** 1 Key Laboratory of Solidification Control and Digital Preparation Technology (Liaoning Province), School of Materials Science and Engineering, Dalian University of Technology, Dalian 116024, China; 18842603762@mail.dlut.edu.cn (H.Z.);; 2 Shanghai Institute of Applied Physics, Chinese Academy of Sciences (CAS), Shanghai 201800, China

**Keywords:** high-entropy alloys, mechanical properties, helium bubbles, irradiation tolerance, low-activation materials

## Abstract

High-entropy alloys (HEAs) have shown promising potential applications in advanced reactors due to the outstanding mechanical properties and irradiation tolerance at elevated temperatures. In this work, the novel low-activation Ti_2_ZrHf_x_V_0.5_Ta_0.2_ HEAs were designed and prepared to explore high-performance HEAs under irradiation. The microstructures and mechanical properties of the Ti_2_ZrHf_x_V_0.5_Ta_0.2_ HEAs before and after irradiation were investigated. The results showed that the unirradiated Ti_2_ZrHf_x_V_0.5_Ta_0.2_ HEAs displayed a single-phase BCC structure. The yield strength of the Ti_2_ZrHf_x_V_0.5_Ta_0.2_ HEAs increased gradually with the increase of Hf content without decreasing the plasticity at room and elevated temperatures. After irradiation, no obvious radiation-induced segregations or precipitations were found in the transmission electron microscope results of the representative Ti_2_ZrHfV_0.5_Ta_0.2_ HEA. The size and number density of the He bubbles in the Ti_2_ZrHfV_0.5_Ta_0.2_ HEA increased with the improvement of fluence at 1023 K. At the fluences of 1 × 10^16^ and 3 × 10^16^ ions/cm^2^, the irradiation hardening fractions of the Ti_2_ZrHfV_0.5_Ta_0.2_ HEA were 17.7% and 34.1%, respectively, which were lower than those of most reported conventional low-activation materials at similar He ion irradiation fluences. The Ti_2_ZrHfV_0.5_Ta_0.2_ HEA showed good comprehensive mechanical properties, structural stability, and irradiation hardening resistance at elevated temperatures, making it a promising structural material candidate for advanced nuclear energy systems.

## 1. Introduction

It is an irresistible trend to obtain clean, low-carbon, and safe energy generated from next generation fission and future fusion energy reactors to meet the needs of human society and industrial development in the long term [1,2,3,4,5]. The absence of compatible structural materials for extreme environments of high temperature, high neutron flux, and chemical reactivity hinders the development of advanced reactors [6,7]. The evolution of microstructures caused by high-energy particles (neutrons, ions, and electrons) irradiation leads to the degradation of mechanical properties, such as embrittlement, hardening, swelling, etc., which eventually threaten the safety and reliability of the reactors [8,9]. Considering one of the significant goals for the advanced reactors is to produce economically clean energy with no long-lived high-radioactivity waste [10,11,12], the low-activation criteria should be included in the structural materials’ design of advanced reactors. Certain achievements have been made in the research of qualified low-activation materials, including oxide dispersion strengthened (ODS) steels [13,14], V-based alloys [15,16], reduced activation ferritic/martensitic (RAFM) steels [17,18], and China low activation martensitic (CLAM) steels [19,20].

In the process of pursuing high-performance materials, the high-entropy alloys (HEAs) [21] were introduced. The preeminent properties (such as high strength [22,23], good corrosion resistance [24,25,26], fine tribological properties [27,28], remarkable softening resistance [29,30], and outstanding irradiation tolerance [1,2,5,31], etc.) enhance the application competitiveness of HEAs under extreme environments. Among them, refractory high-entropy alloys (RHEAs) [32], which are characterized by a high melting point and prepared by methods such as vacuum arc melting, suspension melting, spark plasma sintering [33], and wire electric discharge machining [34], are expected to play a role in future high-temperature applications [35]. Compared with conventional alloys, HEAs, especially the body-centered cubic (BCC) structured ones composed of refractory elements, exhibit better irradiation tolerance for the extreme lattice distortions and chemical complexity, such as prominent resistance to hardening [1,36], suppressed dislocation evolution [11,36,37], low volume swelling rate [38,39], and reduced radiation-induced segregation [40,41,42], etc. Hence, the HEAs are considered to be a promising candidate for nuclear structural materials [1,43] and the corresponding irradiation resistance mechanism has been revealed to a certain extent [2,5,44,45]. The irradiation tolerance of the HEAs could be improved by selecting the appropriate elements and adjusting the chemical complexity [2,46]. Nevertheless, the HEAs designed for nuclear industry applications are limited [36,42].

In this work, by introducing the concept of low-activation materials to the design of HEAs, a novel series of RHEAs with BCC structure were proposed, with the expectation to explore high-performance HEAs under irradiation. In addition, good comprehensive mechanical properties at room and elevated temperatures should also be equipped for the potential application. The basic parameters [47] (melting point (*T*_m_), atomic radius (*r*), density (*ρ*), and valence electron concentration (VEC)) of some commonly used low-activation elements in the nuclear industry are displayed in Table 1. However, not all the elements mentioned are suitable for the design of BCC-structured RHEAs. The high VEC value of Fe element is not conducive to the formation of single-phase BCC structure, and its low melting point could reduce the working temperature of the materials after alloying. The negative mixing enthalpy between Cr and other refractory elements contributes to the appearance of intermetallic compounds, which deteriorates the plasticity of the HEAs. In the as-cast samples, W element with a high melting point is usually seriously segregated, worsening the mechanical and irradiation tolerance. The five refractory elements of Ti, Zr, Hf, V, and Ta were chosen to prepare the low-activation HEAs after considering the basic physical and chemical properties and the alloying characteristics of each element in HEAs. The Ti_2_ZrHf_x_V_0.5_Ta_0.2_ (x values in molar ratio, x = 0.25, 0.5, 0.75 and 1, denoted as Hf0.25Ta, Hf0.5Ta, Hf0.75Ta and Hf1Ta, respectively) low-activation HEAs were designed, and the effects of Hf element on the microstructures and mechanical properties were investigated. As shown in Table 2, according to the phase formation rules of HEAs [48,49,50], all the empirical parameters predicted the formation of single BCC solid solution phase in the Ti_2_ZrHf_x_V_0.5_Ta_0.2_ HEAs.

Ordinarily, the He atoms produced by transmutation reaction would aggregate and form large-scale He bubbles for the limited solubility in the metals, which strongly deteriorated the mechanical properties of the alloys after irradiation [51]. The embrittlement and hardening induced by He atoms were considered as the primary concerns of the stability of structure materials around the half melting temperature (*T*_m_) regime in the nuclear reactors [52]. Therefore, the performance of HEAs under He ion irradiation at elevated temperature is definitely worth investigating. In this work, the evolutions of the microstructures and mechanical properties of the novel low-activation Ti_2_ZrHfV_0.5_Ta_0.2_ HEA with He ions implantation at 1023 K (0.47 *T*_m_) were studied in detail.

## 2. Experimental Section

### 2.1. Materials

The Ti_2_ZrHf_x_V_0.5_Ta_0.2_ HEAs were manufactured by vacuum arc melting under Ar atmosphere. The purity of each raw elemental metal used in this study was higher than 99.9 wt%. All raw metals were purchased from the instrumental and research center of Shanghai Yanku. The furnace chamber was vacuumed to below 5 × 10^−3^ Pa and then protective high-purity argon was reverse charged to 0.05 Pa before starting the melting process. The raw metals were melted on a water-cooled copper crucible. In order to improve the quality of ingots, Ti ingot was melted before melting HEA ingots to remove excess oxygen. For obtaining better homogenization, the ingots were re-melted a minimum of seven times. Each smelting time was 2 min, and the smelting current was approximately 500 A. The acquired samples were button-shaped with a diameter of ~28 mm and a thickness of ~11 mm.

### 2.2. Characterization of Microstructure and Mechanical Properties of As-Cast Samples

The crystal structures of the as-cast samples were characterized by an EMPYREAN X-ray diffractometer (Malvern Panalytical, Almelo, Netherlands) with the 2*θ* scanning from 20 to 100 degrees. The scanning electron microscopy (SEM, Zeiss supra55, ZEISS, Carl Zeiss AG, Jena, Germany) with an energy-dispersive spectrometer (EDS) was introduced to analyze the morphology and chemical compositions. The *Φ* 5 × 10 mm cylindrical specimens prepared by wire electrical discharge machining were used to test the mechanical properties at room temperature (RT) using a Wippermann materials testing machine. A thermal simulation machine of Gleeble-3500 (Data Science International, Sao Paulo City, Brazil) was adopted for the compressive tests at elevated temperature (873 K) with *Φ* 6 × 9 mm cylindrical specimens.

### 2.3. Irradiation Experiment and Characterization of Microstructure of Irradiated Samples

**Figure 1:**
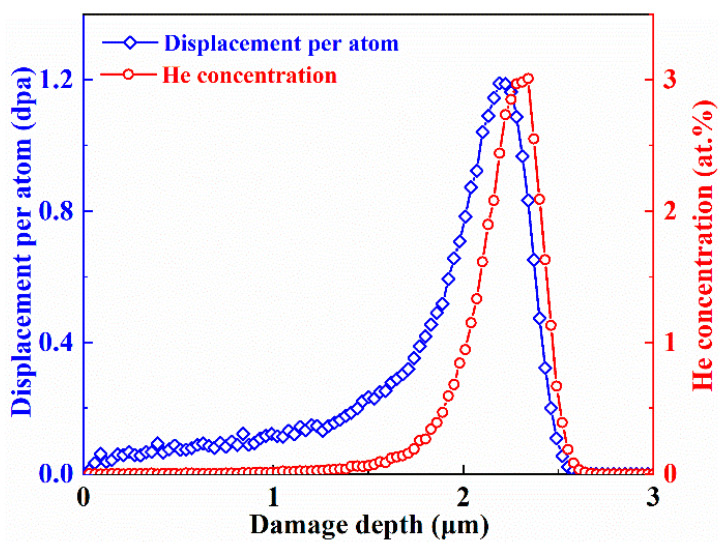
The depth distribution of He concentration and displacement damage in the Hf1Ta HEA at the fluence of 3 × 10^16^ ions/cm^2^.

The irradiation tolerance of the selected representative alloy (Hf1Ta) was identified by He ion irradiation at 1023 K. The prepared specimens were irradiated with 1 MeV He ions to the fluences of 1 × 10^16^ and 3 × 10^16^ ions/cm^2^ at the Shanghai Institute of Applied Physics, Chinese Academy of Sciences (SINAP-CAS, Shanghai, China) using a 4 MV Pelletron accelerator. The sheets (sized 1 mm × 6.5 mm × 10 mm) for the irradiation experiments were taken from the as-cast Hf1Ta HEA. Then, mechanical polishing and electro-polishing were used to optimize the irradiated surface of the samples. At the fluence of 3 × 10^16^ ions/cm^2^, the irradiation damage and He concentration along the depth direction were predicted by Stopping and Range of Ions in Matter (SRIM-2008, http://www.srim.org/, accessed on 3 March 2022), as shown in Figure 1. The displacement energies were set as 30, 40, 90, 40, and 91 eV for the Ti, Zr, Hf, V, and Ta elements, respectively. Simulation results showed that the peak irradiation dose and He concentration were ~1.2 displacements per atom (dpa) and 3.0 at.%, respectively. For observing the microstructures and He bubbles characteristics, the transmission electron microscopy (TEM, Themis Z G3, Thermo Fisher Scientific, Waltham, MA, USA) was employed. Thin films with a thickness of ~60 nm were fabricated by the focused ion beam (FIB, Helios G4UX, Thermo Fisher Scientific, Waltham, MA, USA).

### 2.4. Nanoindentation Test

The hardening behaviors of the samples with He ions implantation were determined by nanoindentation tests (G200 nano-indenter, Technologies, Palo Alto City, CA, USA). More than 8 measurements were adopted to calculate the average hardness of each depth. The size and density of He bubbles in the Hf1Ta HEA were calculated by the Image Pro software (Version 6.0), and more than 2 areas (100 nm × 100 nm) selected from the peak damage regions were chosen to count.

## 3. Results and Discussion

### 3.1. Microstructures of the As-Cast Ti_2_ZrHf_x_V_0.5_Ta_0.2_ HEAs

**Figure 2:**
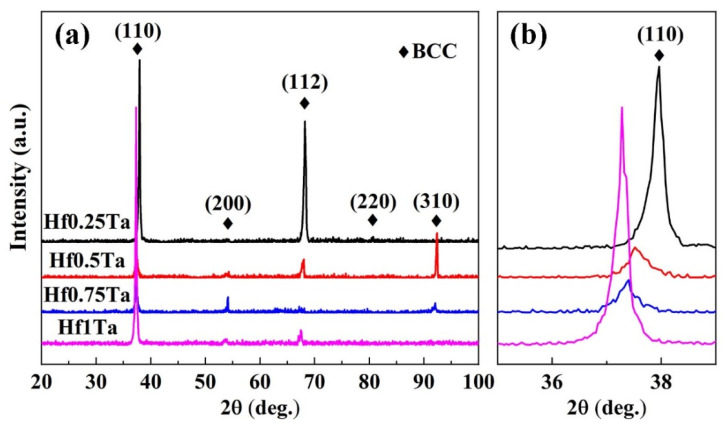
(**a**) The X-ray diffraction patterns of the Ti_2_ZrHf_x_V_0.5_Ta HEAs; (**b**) corresponding detailed scans of the (110) peaks of the BCC phase.

The XRD patterns of the as-cast Ti_2_ZrHf_x_V_0.5_Ta_0.2_ HEAs are exhibited in Figure 2a, in which only the diffraction peaks of BCC phase can be observed. The absence of other phase diffraction peaks in the patterns suggested that the increase of Hf content had little effect on the structure of the Ti_2_ZrHf_x_V_0.5_Ta_0.2_ HEAs.

The shift of the (110) diffraction peaks of BCC phases are displayed in Figure 2b. As can be detected, the (110) peak shifted to a lower 2*θ* angle (decreased from 37.96° in the Hf0.25Ta to 37.28° in the Hf1Ta) as the Hf content increased, implying that tensile strain out-plane was created due the compressive in-plane stress induced by Hf [53,54]. According to Bragg’s law, the values of the lattice constants were calculated to be 0.3349, 0.3387, 0.3398, and 0.3408 nm for Ti_2_ZrHf_x_V_0.5_Ta_0.2_ HEAs corresponding to x = 0.25, 0.5, 0.75, and 1, respectively. The addition of Hf element with the second largest atomic radius could improve the lattice distortion, which contributed to the increase of lattice constants.

**Figure 3:**
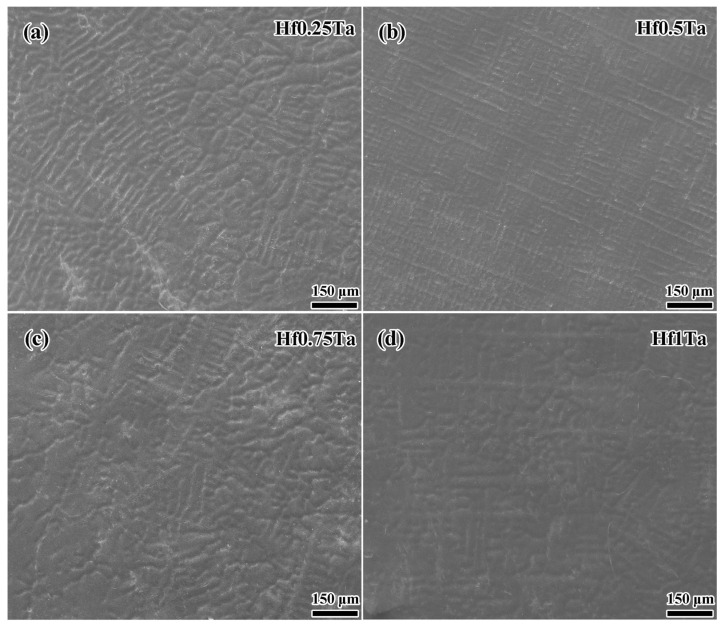
Microstructures of the as-cast (**a**) Hf0.25Ta, (**b**) Hf0.5Ta, (**c**) Hf0.75Ta, and (**d**) Hf1Ta HEAs.

Figure 3 displays the SEM images of the as-cast Ti_2_ZrHf_x_V_0.5_Ta_0.2_ HEAs. All the HEAs exhibited typical dendritic structure and no significant microstructure evolution can be observed in the HEAs with different Hf content. Combined with the XRD results, the Ti_2_ZrHf_x_V_0.5_Ta_0.2_ HEAs exhibited a single BCC solid solution structure. The increase of Hf content could hardly change the microstructures of the Ti_2_ZrHf_x_V_0.5_Ta_0.2_ HEAs significantly, indicating the complete dissolution of Hf element in the matrix. Through EDS analysis, the chemical composition of different regions in the Ti_2_ZrHf_x_V_0.5_Ta_0.2_ HEAs are listed in Table 3. Dendritic regions in the Ti_2_ZrHf_x_V_0.5_Ta_0.2_ HEAs were enriched with higher melting point elements of Hf and Ta elements, while interdendritic regions were enriched with lower melting point elements of Zr and V elements, which could be attributed to the behaviors of elements with different melting points during solidification. It should be noted that the addition of Hf element could mitigate the segregations of the elements and the relatively uniform microstructure was obtained in the Hf1Ta HEA.

### 3.2. Mechanical Properties of the As-Cast Ti_2_ZrHf_x_V_0.5_Ta_0.2_ HEAs

**Figure 4:**
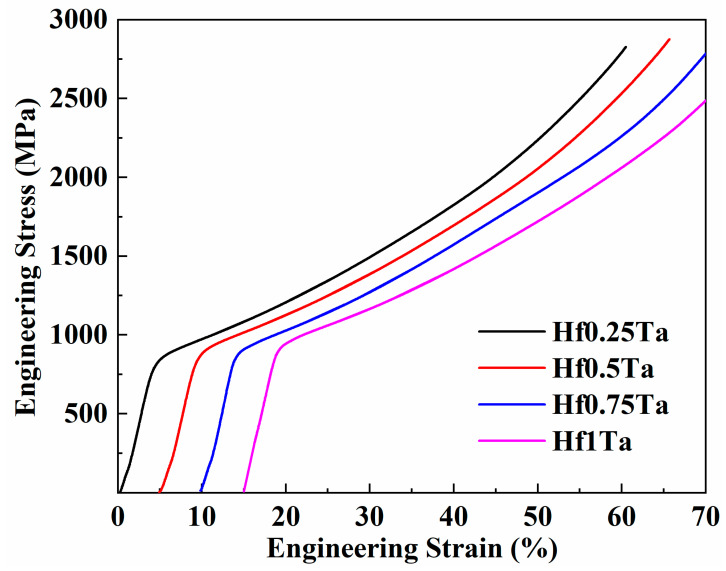
Compressive engineering stress–strain curves of Ti_2_ZrHf_x_V_0.5_Ta_0.2_ HEAs at RT.

Figure 4 exhibits the engineering stress–strain curves of Ti_2_ZrHf_x_V_0.5_Ta_0.2_ HEAs gained by the compression test at RT. The values of yield strength *σ* and plastic strain *ε* are summarized in Table 4. The plastic strain of the Ti_2_ZrHf_x_V_0.5_Ta_0.2_ HEAs was more than 50% and no fracture could be detected during the compression test. Although the changes of Hf content have no obvious effect on the plasticity, the yield strength of the Ti_2_ZrHf_x_V_0.5_Ta_0.2_ HEAs improved from 745 to 873 MPa as the Hf content increased. The advances in mechanical properties were predominantly attributed to the variation in lattice distortion of this series of HEAs. The addition of Hf element with second largest atomic radius intensified the lattice distortion and raised the resistance to dislocation motion, and thus the yield strength was enhanced.

**Figure 5:**
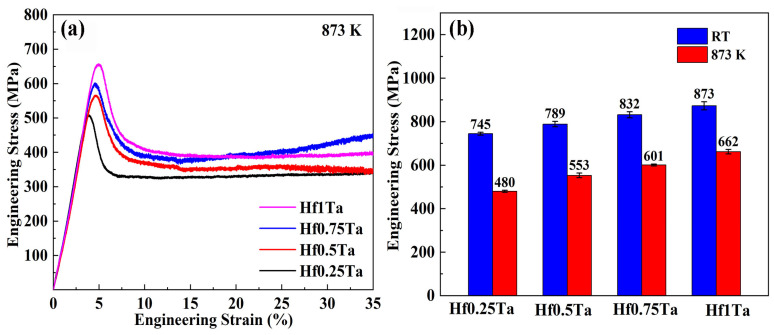
(**a**) Compressive engineering stress–strain curves at 873 K, (**b**) linear graphs of temperature and strength of Ti_2_ZrHf_x_V_0.5_Ta_0.2_ HEAs.

The mechanical properties of the Ti_2_ZrHf_x_V_0.5_Ta_0.2_ HEAs at 873 K have been investigated, and the compressive stress–strain curves are exhibited in Figure 5a. It can be observed from all the flow curves that apparent softening emerged after the appearance of stress peaks at the initial deformation stage, which was a typical manifestation of dynamic recrystallization. The yield strength of the Hf0.25Ta-Hf1Ta HEAs at 873 K was 480, 553, 601, and 662 MPa, respectively. Figure 5b shows the comparison of yield strength of the Ti_2_ZrHf_x_V_0.5_Ta_0.2_ HEAs at different temperatures. As the compression test temperature increased from RT to 873 K, the yield strength decreased by 265, 236, 231, and 211 MPa for the Hf0.25Ta-Hf1Ta HEAs, respectively, and the corresponding decline percentages were 34.4%, 29.9%, 27.8%, and 24.2%, respectively. The compression results indicated that the increase of Hf content played a positive role in enhancing yield strength of the Ti_2_ZrHf_x_V_0.5_Ta_0.2_ HEAs at RT and 873 K. Generally, the alloy melting point was crucial to the softening resistance at elevated temperatures. The melting points of the Ti_2_ZrHf_x_V_0.5_Ta_0.2_ HEAs increased from 2122 K to 2183 K by the increase of Hf content, which was conducive to the softening resistance improvement. On the other hand, the influence of solid solution strengthening caused by the increase of Hf content on the strength cannot be ignored. Under the dual effect, at 873 K the yield strength of Ti_2_ZrHf_x_V_0.5_Ta_0.2_ HEAs improved with the increase of Hf content.

The novel low-activation Ti_2_ZrHf_x_V_0.5_Ta_0.2_ HEAs, especially Hf1Ta HEA, exhibited fine comprehensive mechanical properties at RT and 873 K, which contributed to the industrial application potential in extreme conditions. The He ion irradiation experiment was introduced to preliminarily evaluate irradiation resistance of the Hf1Ta alloy, which was selected as the representative of the designed low-activation Ti_2_ZrHf_x_V_0.5_Ta_0.2_ HEAs due to the high yield strength at RT, elevated temperatures, and high melting point.

### 3.3. TEM Characterization of the Irradiated Ti_2_ZrHfV_0.5_Ta_0.2_ HEA

**Figure 6:**
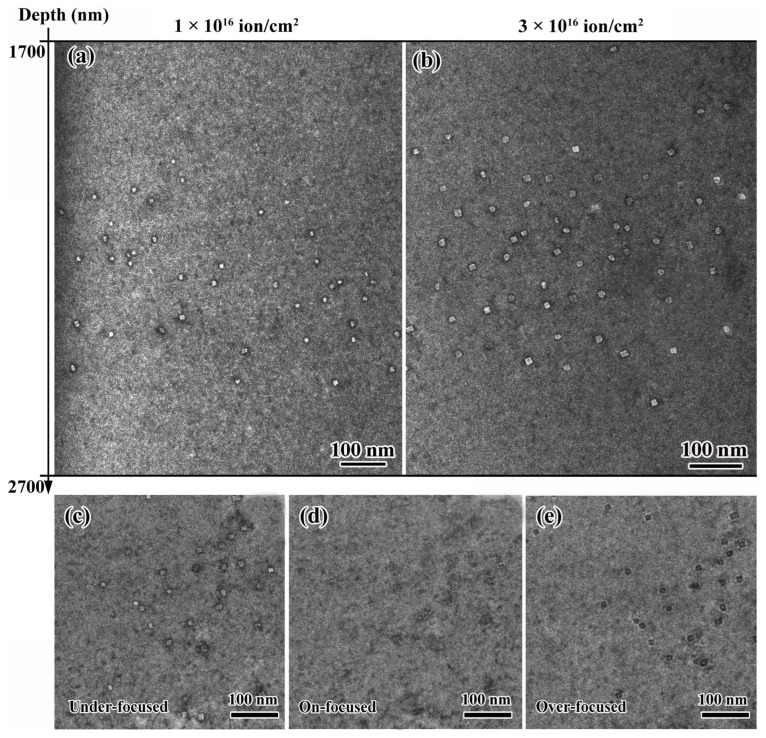
(**a**,**b**) Distribution of He bubbles at 1700~2700 nm depth in irradiated samples at different fluences; (**c**–**e**) the under-, on-, and over-focused images of He bubble characterization obtained at the same position.

The characterizations of He bubbles, such as distribution range, shape, size, and number density, were primarily analyzed in this work. Based on the SRIM simulation results (shown in Figure 1), the peaks of irradiation damage and He concentration emerged at the depth of ~2200 and 2300 nm, respectively. Therefore, at different fluences, the cross-sectional TEM images containing peak damage regions at depths of 1700 to 2700 nm are shown in Figure 6a,b, presenting the He bubbles’ distribution characterizations. For the sample irradiated to a fluence of 1 × 10^16^ ions/cm^2^, the He bubbles emerged at the depth of ~2000 nm and extended to 2500 nm, and no bubbles could be identified beyond this range. A wider spatial distribution of He bubbles was detected in the sample irradiated to a higher fluence of 3 × 10^16^ ions/cm^2^. As can be detected in Figure 6b, larger bubbles were observed in the depth range of 1850~2550 nm, which was roughly consistent with the simulation results shown in Figure 1.

The features of He bubbles in the TEM bright field images taken in the same position under different focusing states are presented in Figure 6c–e, in which white and black spots were found in under- and over-focused conditions, respectively. Under different focusing states, no precipitates could be observed in the peak damage regions.

**Figure 7:**
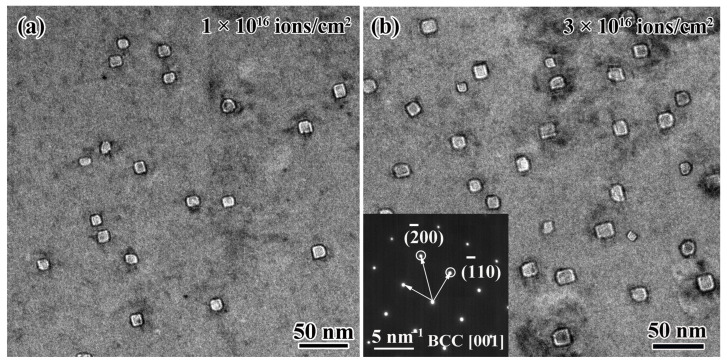
Comparison of He bubble characterizations in the peak damage regions of the Hf1Ta HEA at the fluences of (**a**) 1 × 10^16^ and (**b**) 3 × 10^16^ ions/cm^2^.

The characterizations of the He bubbles at high magnification are displayed in Figure 7a,b, in which faceted bubbles were observed in the Hf1Ta alloy at different fluences. The He bubbles in BCC-structured conventional materials usually evolved into polygons at elevated temperatures to maintain a more stable state and faceted bubbles formed in the Nb-Zr and Fe-Cr alloys [55]. The shape of He bubbles in materials can be influenced by several factors, including crystal structure, surface energy, strain effects, volume energy, etc. The extra elastic strain energy was generated by vacancy and He atoms flowing into He bubbles at high temperatures. For maintaining a more stable state of He bubbles, large areas of surfaces were formed and developed on low-energy planes [56], which resulted in the formation of faceted bubbles in the Ti_2_ZrHf_x_V_0.5_Ta_0.2_ HEA at 1023 K. The morphology of the bubbles in the Hf1Ta alloy in this study, Ti-Zr-Nb-V-Mo [46], and Ti-V-Nb-Ta RHEAs [57] was similar to that found in the conventional materials with He ions implantation, suggesting that the formation of faceted bubbles at elevated temperature may be a feature of BCC-structured RHEAs.

**Figure 8:**
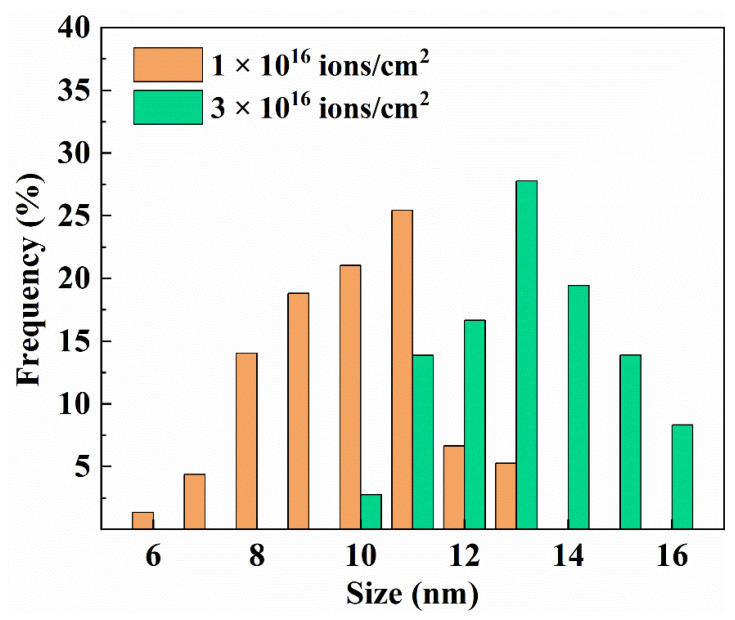
Size distributions of He bubbles in the Hf1Ta HEA at different fluences.

At different fluences, no precipitations could be found in the peak damage regions of the irradiated Ha1Ta HEA and only the diffraction spots of BCC phase were detected in the selected area’s electron diffraction patterns obtained from different irradiation damage regions, and they suggested preeminent phase stability of the Hf1Ta HEA under He ion irradiation at 1023 K. The bubbles’ distributions in peak damage regions were determined to be random and uniform under different fluences, and the He bubble sizes presented normal-like distributions (as displayed in Figure 8), meaning that the nucleation and growth process of He bubbles in the Hf1Ta HEA was homogeneous under irradiation [57].

**Figure 9:**
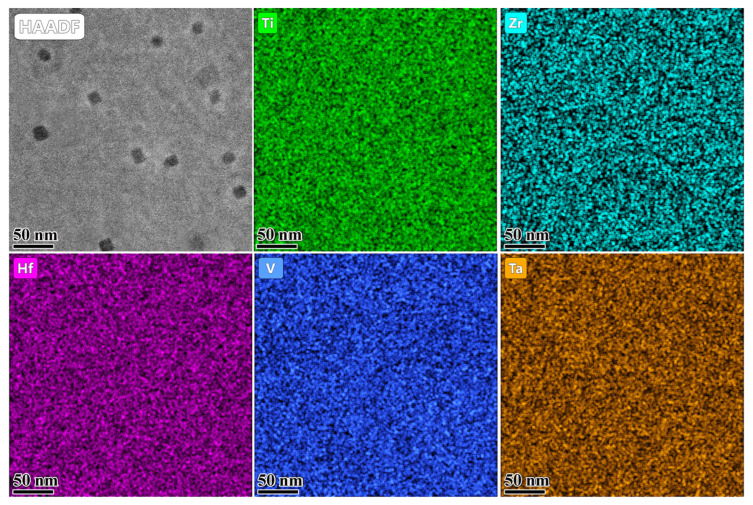
Elemental distribution near the He bubbles in the Hf1Ta HEA irradiated to the fluence of 3 × 10^16^ ions/cm^2^.

Figure 9 exhibits the elemental distributions near the bubbles in the Hf1Ta HEA irradiated to a fluence of 3 × 10^16^ ions/cm^2^. No obvious elemental enrichment regions were detected in the images and the distribution of each element was relatively uniform. Recent studies [40,57] on the HEAs indicated that the atomic size difference dominated the elements segregation under irradiation. In those cases, the vacancies away from the He bubbles were preferentially coupled with the oversized elements, resulting in the enrichment of the undersized elements at the bubbles. Significantly, the radiation-induced segregation may affect the behavior of dislocations/He bubbles and induce stress corrosion cracking, thereby degrading the mechanical properties and putting a negative impact on the irradiation performance of the alloys. The uniform distribution of elements near the He bubbles suggested the good structural stability of the Hf1Ta HEA under the given irradiation condition.

The size distributions of the He bubbles in the peak damage regions are shown in Figure 8. At each fluence, the He bubble sizes in the Hf1Ta HEA presented a unimodal distribution. The average sizes (*d*) and number densities (*N*) of the He bubbles in the Hf1Ta HEA at different fluences are listed in Table 5. The average size increased from 10.5 nm to 13.7 nm and number density increased from 9.09 × 10^20^ m^−3^ to 2.42 × 10^21^ m^−3^ as the fluence improved. 

Generally, the growth of He bubbles was temperature sensitive and larger He bubbles were found at elevated temperature [58]. The average sizes of He bubbles in the conventional materials were summarized in Table 6. Owing to the limitation of melting point, the temperature of irradiation experiments applied to conventional materials was restricted, which was no more than 973 K. Therefore, the He bubble sizes in the Hf1Ta HEA were larger than those in conventional materials [59,60,61,62,63] due to the higher experiment temperature (1023 K), while He bubble density was one to two orders of magnitude lower. Significantly, the He bubble sizes in the Hf1Ta HEA were between 5–17 nm, which were close to those found in the reported Ti-Zr-Nb-V-Mo [46] at 1023 K and Ti-V-Nb-Ta RHEAs [57] at 973 K. However, the average sizes of He bubbles in tens of nanometers (34.1–85.6 nm) were found in FCC-structured NiCoFeCrMn HEAs and its derivatives [58] at 973 K.

### 3.4. Irradiation Hardening

**Figure 10:**
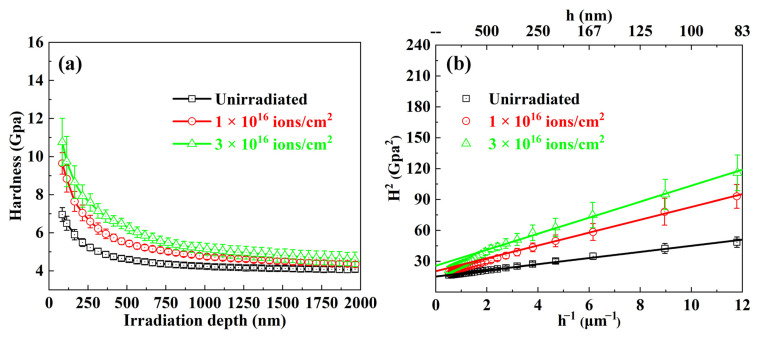
(**a**) The average nano-hardness measurements as a function of depth of the unirradiated and irradiated Hf1Ta HEA; (**b**) corresponding profiles of *H*^2^ versus *h*^−1^.

Nano-indentation tests were employed to assess the mechanical properties of the Hf1Ta HEA after He ions’ irradiation. Figure 10a shows the average hardness dependence of indentation depths of the unirradiated and irradiated Hf1Ta alloy. Due to the surface effect on the measurement accuracy, hardness values measured at the depths less than 80 nm were not reliable and omitted. At the same depth, the irradiated sample possessed higher hardness values than the unirradiated one, which suggested the hardening of irradiated Hf1Ta HEA. For the indentation size effect, the hardness decreased slightly with increasing the indentation depth as shown in Figure 10a, which could be described by the model proposed by Nix and Gao [61]:

(1) $$H=H_{0}\sqrt{1+(h^{*}/h)}$$

where *H* represents the measured hardness, *H*_0_ represents the hardness at infinite depth, h* represents a characteristic length which depends on the material and the shape of indenter tips, and *h* represents the indentation depth.

For the uniform material, such as the unirradiated alloys, there was no deviation away from linearity in the profiles of *H*^2^ versus *h*^−1^ based on the Nix–Gao model. As shown in Figure 10b, the hardness curves of the unirradiated Hf1Ta HEA exhibited a good linear relationship in the irradiation depth range of 80–2000 nm. The irradiated alloy was heterogeneous, with a hard and thin damaged layer near the surface. The volume of the plastic zone generated by the indenter pressing into the surface of the sample is usually much larger than that of the indenter. As the plastic zone extended to the lower undamaged region, the measured hardness value could be affected. In this case, the measured hardness value decreased with the increase of depth and finally approached the hardness of the unirradiated area, which was named as softer substrate effect (SSE) [61]. Therefore, there was a deviation away from linearity when the depth value was large enough (deeper than ~350 nm for the Hf1Ta HEA in this study). The values corresponding to the linearity were usually used for fitting to compare and study the hardness changes of the alloy before and after irradiation to a certain extent. In this study, due to indentation size effect and soft substrate effect, the values of hardness measured in the depth range of 80–350 nm in the irradiated Hf1Ta HEA and 80–2000 nm in the unirradiated Hf1Ta HEA were used for fitting to evaluate the hardness change.

The hardness value of the unirradiated sample was calculated as 3.78 GPa and the hardness increment (Δ*H*, hardness difference between unirradiated and irradiated samples) and hardening fraction (Δ*H*/*H*_0_, the ratio of the hardness increment to the hardness of unirradiated sample) are summarized in Table 7. As the irradiation fluence increased from 1 × 10^16^ to 3 × 10^16^ ions/cm^2^, the hardness increment increased from 0.67 to 1.34 GPa, and the hardening fraction increased from 17.7% to 34.1%. Remarkably, the irradiation hardening fraction of the Hf1Ta HEA was equivalent to that of the BCC structured Ti-Zr-Nb-V-Mo HEAs, which was lower than those of most reported conventional low-activation materials at similar He ions’ irradiation fluences (shown in Table 8). RHEAs of different systems showed good irradiation hardening resistance under He ion irradiation, and the selected elements could greatly affect the irradiation resistance of the HEAs, which is worthy of further study in the future.

Irradiation-induced defects, including dislocation loops, He bubbles, and stacking faults, could act as the barrier of sliding dislocations, and have a significantly negative impact on the mechanical properties of the irradiated alloys. It was reported that the obstacle strength of He bubble defects was strongly related to their sizes [66,67,68]. Compared to the smaller defects, the probability of interaction with dislocations was enhanced due to the large cross section of the oversized bubbles [46]. The maximum He bubble size in Hf1Ta HEA can reach more than 16 nm with average sizes of 10.5 and 13.7 nm at different fluences, which was conducive to enhancing the interaction between He bubbles and dislocations and improving the hardness. Thus, the formation of large He bubbles in the irradiated Hf1Ta HEA could be considered as the main cause of hardening. Compared with the sample irradiated at low fluence, the larger and denser He bubbles generated in the sample irradiated at high fluence can interact with the dislocations more effectively, which led to the higher hardness increment and hardening fraction in the Hf1Ta HEA irradiated at the fluence of 3 × 10^16^ ions/cm^2^. Additionally, Zhao [33] and Shi [34] proposed that residual density of vacancies and interstitials produced by irradiation could be greatly reduced by the effective recombination in the HEAs. The severe lattice distortion in HEAs could impose restrictions on the formation and growth of defect clusters, resulting in small size defects with low density in the matrix. For those reasons, the force on dislocation movement could be weakened and the degradation of mechanical properties of the Hf1Ta HEA after irradiation was mitigated. Fine structural stability, limited radiation-induced segregation, and low residual defect density contributed to the good irradiation tolerance of the Hf1Ta HEA.

## 4. Conclusions

The novel low-activation Ti_2_ZrHf_x_V_0.5_Ta_0.2_ HEAs were designed and prepared. He ion irradiation experiments were employed to preliminarily evaluate irradiation tolerance of the representative Hf1Ta HEA. The microstructures and mechanical properties of the as-cast and irradiated samples were investigated. The main conclusions are as follows:

(1) The as-cast Ti_2_ZrHf_x_V_0.5_Ta_0.2_ HEAs exhibited BCC solid solution structure and the plastic strain exceeded 50%. Due to the solid solution strengthening caused by the increase of Hf content, the yield strength of the Ti_2_ZrHf_x_V_0.5_Ta_0.2_ HEAs enhanced from 745 to 873 MPa at room temperature and from 480 to 662 MPa at 873 K.
(2) No obvious radiation-induced element segregations or precipitations were found in the He-implanted Hf1Ta HEA, which reflected fine structural stability under He ion irradiation at 1023 K.
(3) The morphology of the He bubbles in the Hf1Ta HEA was faceted, which was similar to that found in the BCC structured conventional materials, Ti-Zr-Nb-V-Mo, and Ti-V-Nb-Ta RHEAs at elevated temperatures.
(4) As the irradiation fluence increased from 1 × 10^16^ to 3 × 10^16^ ions/cm^2^, the average size of the He bubbles in Hf1Ta HEA increased from 10.5 to 13.7 nm and number density increased from 9.09 × 10^20^ to 2.42 × 10^21^ m^−3^.
(5) With improving fluence, the irradiation hardness increment increased from 0.67 to 1.34 GPa, and the hardening fraction increased from 17.7% to 34.1%. Due to the low residual defect density and fine structural stability, the hardening fraction of the irradiated Hf1Ta HEA was lower than those of most reported conventional low-activation materials at similar He ions’ irradiation fluences. The experimental results indicated that the novel low-activation RHEA may be one of the promising candidate structural materials for advanced nuclear energy system.

## Figures and Tables

**Table 1:** The values of *T*_m_ (K), *r* (nm), *ρ* (g/cm^3^), and VEC of some reduced-activation elements.

Elements	Fe	Ti	Zr	Cr	V	Hf	Ta	W
*T*_m_ (K)	1811	1941	2128	2180	2183	2506	3290	3695
*r* (nm)	0.126	0.147	0.160	0.128	0.134	0.159	0.146	0.139
*ρ* (g/cm^3^)	7.87	4.51	6.51	7.14	6.11	13.31	16.65	19.25
VEC	8	4	4	6	5	4	5	6

**Table 2:** Values of VEC, *δ* (%), *Ω*, Δ*H*_mix_ (kJ mol^−1^), and Δ*S*_mix_ (J K^−1^ mol^−1^) of the Ti_2_ZrHf_x_V_0.5_Ta_0.2_ HEAs.

Alloys	VEC	Δ	*Ω*	Δ*H*_mix_	Δ*S*_mix_
Hf0.25Ta	4.18	5.51	27.52	−0.82	10.64
Hf0.5Ta	4.17	5.54	32.09	−0.83	11.20
Hf0.75Ta	4.16	5.54	36.17	−0.69	11.48
Hf1Ta	4.15	5.52	283.96	−0.63	11.60

**Table 3:** Chemical composition of dendritic (D) and interdendritic (ID) regions of the Ti_2_ZrHf_x_V_0.5_Ta_0.2_ HEAs in atomic percentage.

Alloys	Regions	Ti	Zr	V	Hf	Ta
Hf0.25Ta	D	45.65	24.43	9.76	14.04	6.12
	ID	45.35	25.39	11.83	12.96	4.47
Hf0.5Ta	D	45.18	23.50	9.70	15.81	5.81
	ID	45.31	24.02	10.56	14.72	5.40
Hf0.75Ta	D	44.11	22.39	9.53	18.37	5.60
	ID	44.02	22.80	10.31	17.60	5.26
Hf1Ta	D	42.24	21.01	9.04	23.26	4.45
	ID	42.42	21.53	9.58	22.54	4.11

**Table 4:** Values of melting temperature and mechanical properties of the Ti_2_ZrHf_x_V_0.5_Ta_0.2_ HEAs at RT.

Alloys	*σ* (MPa)	*ε* (%)	*T*_m_ (K)
Hf0.25Ta	745	>50	2122
Hf0.5Ta	789	>50	2145
Hf0.75Ta	832	>50	2165
Hf1Ta	873	>50	2183

**Table 5:** Average size and density of He bubbles in the Hf1Ta HEA at different fluences.

Fluence (ions/cm^2^)	Average Size (nm)	Number Density (×10^20^ m^−3^)
1 × 10^16^	10.5	9.09
3 × 10^16^	13.7	24.21

**Table 6:** Average sizes of He bubbles in the conventional materials (CMs) and typical HEAs and their derivatives at elevated temperatures.

Alloys		Temperature (K)	Fluence (ions/cm^2^)	Peak He Concentration (at.%)	Average Size (nm)
HEAs	TiVTa [57]	973	1 × 10^17^	5.0	13.4
	TiVNbTa [57]	973	1 × 10^17^	5.0	8.1
	TiZrNbV [46]	1023	6 × 10^16^	3.9	12.5
	TiZrNbVMo [46]	1023	6 × 10^16^	3.9	10.4
	NiCo [58]	973	6.4 × 10^16^	3.6	25.1
	NiCoCr [58]	973	6.4 × 10^16^	3.6	34.1
	NiCoFeCrMn [58]	973	6.4 × 10^16^	3.6	85.6
CMs	V-4Cr-4Ti [59]	573	5 × 10^16^	4.0	2.7
	ODS [60]	723	1 × 10^17^	5.6	3.9
	RAFM [63]	773	3 × 10^16^	5.7	5.1
	GH3535 [62]	923	1 × 10^17^	5.0	2.3
	Ni-SiC [64]	923	6 × 10^16^	3.5	8.1

**Table 7:** Nanoindentation test results of the Hf1Ta HEA at different fluences.

Fluence (ions/cm^2^)	*H*_0_ (GPa)	Δ*H* (GPa)	Δ*H/H*_0_ (%)
0	3.78	-	-
1 × 10^16^	4.45	0.67	17.7
3 × 10^16^	5.07	1.34	34.1

**Table 8:** The hardening fraction of some He-irradiated conventional low-activation materials and HEAs.

Alloys		Temperature (K)	Fluence (×10^16^ ions/cm^2^)	Hardening Fraction (%)
HEAs	TiZrNb [46]	1023	6	17.3
	TiZrNbV [46]	1023	6	41.3
	TiZrNbVMo [46]	1023	6	23.6
CMs	ODS [60]	723	5	48.1
	V-Cr-Ti [59]	573	5	52.0
	RAFM [65]	773	3	85.9
	CLAM [19]	773	3	61.1

## Data Availability

Not applicable.
